# miR-493 mediated DKK1 down-regulation confers proliferation, invasion and chemo-resistance in gastric cancer cells

**DOI:** 10.18632/oncotarget.6951

**Published:** 2016-01-20

**Authors:** Xiaoting Jia, Nan Li, Cong Peng, Yingen Deng, Jia Wang, Min Deng, Minying Lu, Jiang Yin, Guopei Zheng, Haiying Liu, Zhimin He

**Affiliations:** ^1^ Cancer Research Institute and Cancer Center of Guangzhou Medical University, Guangzhou Key Laboratory of “Translational Medicine on Malignant Tumor Treatment”, Guangzhou 510095, Guangdong, China; ^2^ Department of Gastrointestinal Neoplasms Surgery, Cancer Center of Guangzhou Medical University, Guangzhou Key Laboratory of “Translational Medicine on Malignant Tumor Treatment”, Guangzhou 510095, Guangdong, China

**Keywords:** miR-493, DKK1, gastric cancer, proliferation, invasion

## Abstract

In the present study, we demonstrated that the levels of DKK1 were decreased in serums and tissues of GC. DKK1 levels inversely correlated with tumor class, TNM stage, distant metastasis and lymph node metastasis of GC. GC patients with low DKK1 levels had a poor overall survival. DKK1 inhibited the proliferation of GC cells *in vitro* and *in vivo*. DKK1 also inhibited invasion, but enhanced chemo-sensitivity of GC cells. Mechanically, miR-493 levels increased in GC and directly targeted and down-regulated DKK1 expression. In agreement, miR-493 promoted proliferation of GC cells *in vitro* and *in vivo*. MiR-493 also promoted invasion and chemo-resistance of GC cells. However, DKK1 overexpression reversed the effects of miR-493 on proliferation, invasion and chemo-sensitivity. Thus, our results provide new insight for the role of miR-493/DKK1 axis in GC.

## INTRODUCTION

Gastric cancer (GC) is the fourth most common malignant disease worldwide and the secondly main cause of death from cancer [1]. There was an incidence of 989,000 cases and a mortality of 735,000 cases for GC in 2011 [2, 3]. In spite of advances in developing more efficient surgical techniques and novel chemotherapeutic interventions, the long term survival rate of GC patients remains poor. Chemo-resistance and invasion are still the major obstacles for successful GC treatment. The precise mechanisms for progression in GC remain elusive.

Dickkopf-1 (DKK1), a secreted protein, usually is reported to interact with the LRP5/LRP6 co-receptor, to inhibit the canonical Wnt/β-catenin signaling [4, 5]. DKK1 plays a diverse roles in different cancers [6]. Some reports discovered that DKK1 was overexpressed in multiple myeloma, hepatoblastomas, Wilms' tumors, breast cancer and hepatocellular carcinoma (HCC), and functioned as a tumor promoter [7–10]. On the other hand, several studies supported that DKK1 suppressed tumors growth [11–13]. The tumor suppressor function of DKK1 has been demonstrated in a colon cancer model, and epigenetic silence of DKK1 has been reported in colorectal cancers [14]. In regard to roles of DKK1 in GC, there are also converse reports. Report from Gao *et al.* showed that DKK1 was overexpressed in GC [15], but investigation from Cai *et al.* demonstrated that DKK1 could reduce the self-renewal capacity of cancer stem-like cells in GC via blocking the wnt/β-catenin signal [16]. Thus, it is urgent to clarify the role of DKK1 in GC and the mechanisms for DKK1 deregulation.

Cancer tissue samples are the best for evaluating cancer markers, but blood samples are more readily available and require a relatively noninvasive procedure. Recently, an increased DKK1 serum level has been reported to be useful as a cancer biomarker in lung, esophageal, cervical, and endometrial cancers [17, 18]. However, these findings about the serum levels of DKK1 were still controversial. Serum DKK1 was found to be elevated in cancer types such as HCC, breast cancer with bone metastasis and cervical cancer [19–21]. In contrast, serum DKK1 was also reported to be significantly lower in cancers, such as in multiple myeloma responding to anti-myeloma treatment [22], clear cell renal cell carcinoma [23] and so on. These controversial results indicated that DKK1 should further be studied to distinct its roles in cancers. The mechanisms for DKK1 deregulation also remains to be elusive.

In this study, we found that DKK1 expression was markedly decreased in GC tissues and serum samples. DKK1 expression inversely correlated with miR-493 levels and is a direct target of miR-493. Moreover, miR-493 modulated the proliferation, invasion and chemo-sensitivity of GC cells via suppressing DKK1 expression.

## RESULTS

### DKK1 levels decreased in GC, and associates with prognosis of GC patients

**Figure 1:**
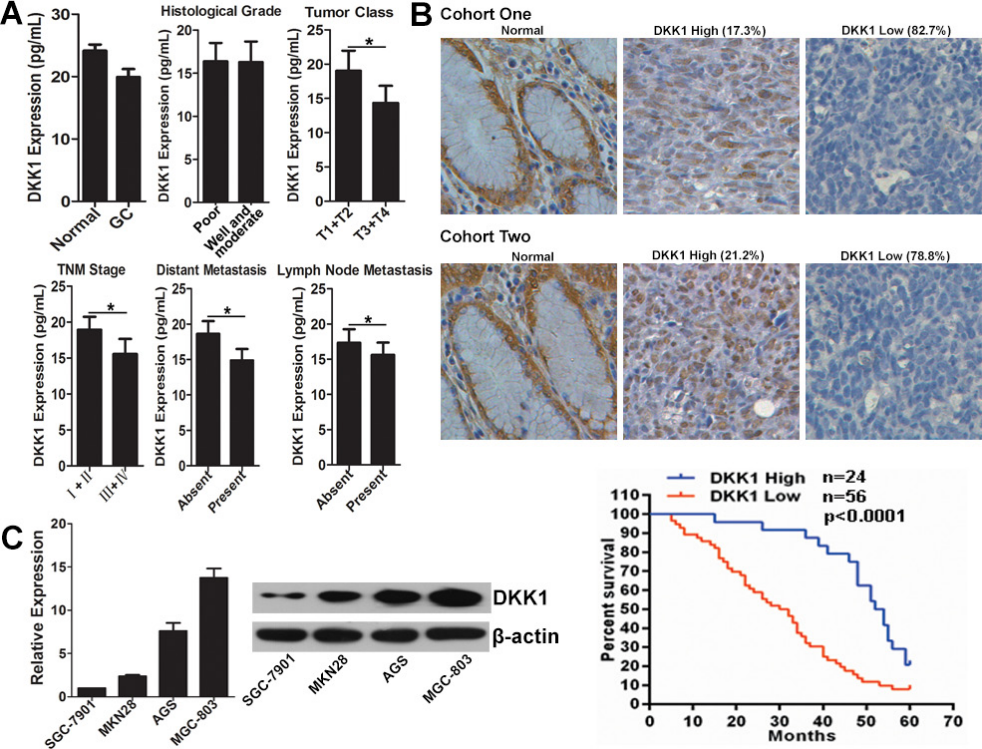
DKK1 expression pattern in GC (**A**) The serum level of DKK1 protein in normal people and GC patients were measured by ELISA assay. Meanwhile, the distribution of DKK1 in serum of GC patients with different clinicopathologic parameters was also analyzed. *p < 0.01. (**B**) Representative images of DKK1 protein level detected by immunohistochemical staining in GC tissues matched of serum samples, and in GC tissues of patients with follow-up information (20×). Kaplan-Meier analysis estimated overall survival according to the DKK1 level in GC patients. (**C**) mRNA and protein expression levels of DKK1 in GC cell lines were evaluated by real-time RT-PCR and western blot, respectively.

To explore the potential role of DKK1 in GC, we first detected its expression pattern in GC serum. As shown in Figure 1A, the DKK1 protein level in GC serum was significantly reduced comparing with normal control. In the 116 GC patients, the DKK1 level inversely correlated with clinical parameters, such as tumor class, TNM stage, distant metastasis and lymph node metastasis, but not the histological grade (Figure 1A, Table 1). Meanwhile, we conducted immunohistochemistry to evaluate DKK1 protein level in 34 cases of normal and 132 cases of GC tissues. The GC tissues were separated into two cohorts. For cohort one, 52 tissues were obtained from GC patients also enrolled for their DKK1 serum concentrations. The results indicated that DKK1 expression was down-regulated in 82.7% GC tissues, which was in accordance with that in serum (Figure 1B). For cohort two, 80 GC tissues were got with follow-up information. We found DKK1 was down-regulated in 78.8% GC tissues compared with normal tissues in cohort two (Figure 1B). To assess the significance of DKK1 in terms of clinical prognosis, Kaplan–Meier survival analysis was conducted among the 80 GC tissues. The results showed that patients with low DKK1 expression had a poorer overall survival than the patients with high DKK1 expression in cohort two (Figure 1B). Additionally, DKK1 expression level in GC cells was determined by real-time RT-PCR and western blot. It was indicated that DKK1 is relatively highly expressed in MGC-803 cell with low-invasive ability, and is lowly expressed in high-invasive GC cell line SGC-7901 (Figure 1C). These results demonstrated that DKK1 is down-regulated in GC and its down-regulation negatively correlated with prognosis.

**Table 1:** Analysis of the correlation between expression of DKK1 and miR-493 in GC and its clinicopathologic parameters

Viable	Cases	DKK1		miR-493	
		Expression (pg/mL)	*P* value	Expression	*P* value
**Histological Grade**					
Poor	81	16.383 ± 2.130	0.784	3.985 ± 0.784	0.599
Well and moderate	35	16.277 ± 2.402		4.062 ± 0.920	
**Tumor Class**					
T1 + T2	50	19.025 ± 2.938	0.000	3.655 ± 1.163	0.000
T3 + T4	66	14.352 ± 2.482		5.584 ± 1.599	
**TNM Stage**					
I + II	27	18.935 ± 1.798	0.000	3.669 ± 0.989	0.000
III + IV	89	15.556 ± 2.108		4.837 ± 1.387	
**Distant Metastasis**					
Absent	45	18.645 ± 1.788	0.000	3.584 ± 0.939	0.000
Present	71	14.883 ± 1.609		5.187 ± 1.272	
**Lymph Node Metastasis**					
Absent	49	17.347 ± 1.905	0.000	3.990 ± 1.227	0.000
Present	67	15.622 ± 1.747		4.843 ± 1.357	

### DKK1 inhibits proliferation, invasion and chemo-resistance of GC cells

**Figure 2:**
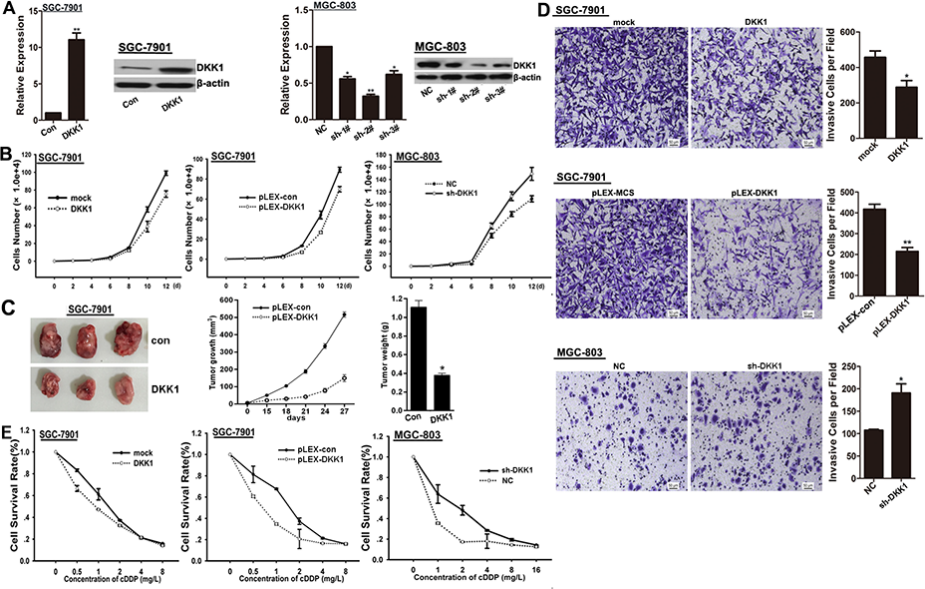
DKK1 impairs proliferation, invasion and chemo-resistance of GC cells (**A**) mRNA and protein expression levels of DKK1 were detected in SGC-7901 cells transfected with DKK1 overexpressing plasmid and in MGC-803 cells transfected with shRNAs against DKK1. Finally, sh-#2 was selected for the further experiments. vs related control, *p < 0.05, **p < 0.01. (**B**) The role of DKK1 on cell proliferation was determined and the average cell number of each time point was indicated. (**C**) Tumor growth was monitored every 3 days. Tumor size and weight were recorded. Data are represented as a mean ± SD from three mice. *vs* control, *p < 0. 01. (**D**) The invasive potential was determined by transwell assay. vs related control, *p < 0.05, **p < 0.01. (**E**) DKK1 overexpression enhanced the sensitivity of GC cells to cDDP, but DKK1 knockdown attenuated the chemo-sensitivity of GC cells.

According to the results of Figure 1C, we performed over-expressed DKK1 in SGC-7901 cells, and knocked-down DKK1 via shRNAs in MGC-803 cells (Figure 2A). We found that cultured with exogenous DKK1 protein or over-expressed by transfection with pLEX-DKK1 plasmid could remarkably impair the proliferation potential of SGC-7901 cells comparing to the control treated cells, while the opposite results were got in MGC-803 cells after DKK1 knockdown with psi-LVRU6GP-shDKK1 plasmid against DKK1 (Figure 2B). Moreover, the effect of DKK1 on growth in GC cells was further examined in nude mice with GC xenografts. We injected subcutaneously into the oxter of Athymic mice with DKK1 overexpressing SGC-7901 cells and their control cells. As shown, tumors derived from DKK1 overexpressing SGC-7901 cells had a significantly reduced tumor size compared with related control cells (Figure 2C). Additionally, both DKK1 treatment and DKK1 overexpression led to a robustly invasion inhibition in SGC-7901 cells, but DKK1 knockdown enhanced the invasion potential of MGC-803 cells (Figure 2D). As shown in Figure 2E, both DKK1 treatment and DKK1 overexpression sensitized SGC-7901 cells to cDDP, whereas, DKK1 knockdown enhanced chemo-resistance of MGC-803 cells to cDDP. In view of these data, we proposed that DKK1 might be involved in proliferation, invasion and chemo-sensitivity of GC cells.

### miR-493 suppresses DKK1 expression in GC cells

**Figure 3:**
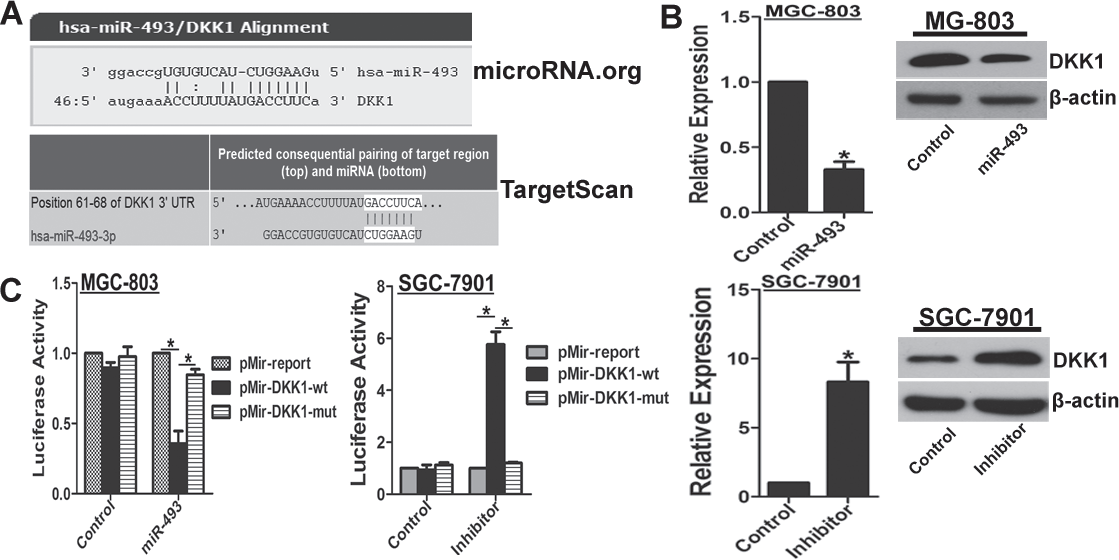
miR-493 directly targets DKK1 (**A**) The assumed regulating microRNAs of *DKK1* were predicted by online software microRNA.org and TargetScan. Schematic diagram of putative miR-493 binding site in the 3′UTR regions of *DKK1* was showed. (**B**) Functional interference of miR-493 negatively regulated DKK1 expression in mRNA and protein levels determined by real-time RT-PCR and western blot. *vs* control, *p < 0. 01. (**C**) Functional interference of miR-493 negatively associated with the activity of luciferase gene linked with the 3′UTR sequence of *DKK1* and a renilla luciferase reporter was used for normalization. Luciferase activities were measured at 48 hours after transfection and the data was obtained from three independent experiments. The mean of the results from negative control reporter and interference control were set as 1. *p < 0.01.

To explore the mechanism for DKK1 down-regulation, here, potential miRNAs targeting *DKK1* were predicted with online software microRNA.org and TargetScan, respectively (Figure 3A). To investigate whether miR-493 directly targets *DKK1*, we found increased miR-493 via transfection of miR-493 expressing plasmid pEZX-miR-493 significantly down-regulated DKK1 mRNA and protein levels in MGC-803 cells (Figure 3B). Meanwhile, miR-493 inhibitor clearly up-regulated DKK1 mRNA and protein levels in SGC-7901 cells (Figure 3B). Furthermore, we constructed luciferase reporter vectors with the putative *DKK1* 3′-UTR target site for miR-493 at downstream of the luciferase gene (pMir-DKK1-wt, set as wild-type) and mutant version with deletion of 7bp in the seed sequence (pMir-DKK1-mut). Results of reporter gene assays demonstrated that miR-493 overexpression observably decreased luciferase activity of pMir-DKK1-wt vector, but the mutant version abrogated the suppressive ability of miR-493 overexpression in MGC-803 cells (Figure 3C). Inversely, miR-493 inhibitor evidently enhanced luciferase activity of pMir-DKK1-wt vector, and the mutant version abrogated the promotive effect of miR-493 inhibitor in SGC-7901 cells (Figure 3C). These findings strongly suggested that DKK1 is a novel direct target of miR-493 in GC cells.

### miR-493 is up-regulated in GC and works as a promoter in GC

**Figure 4:**
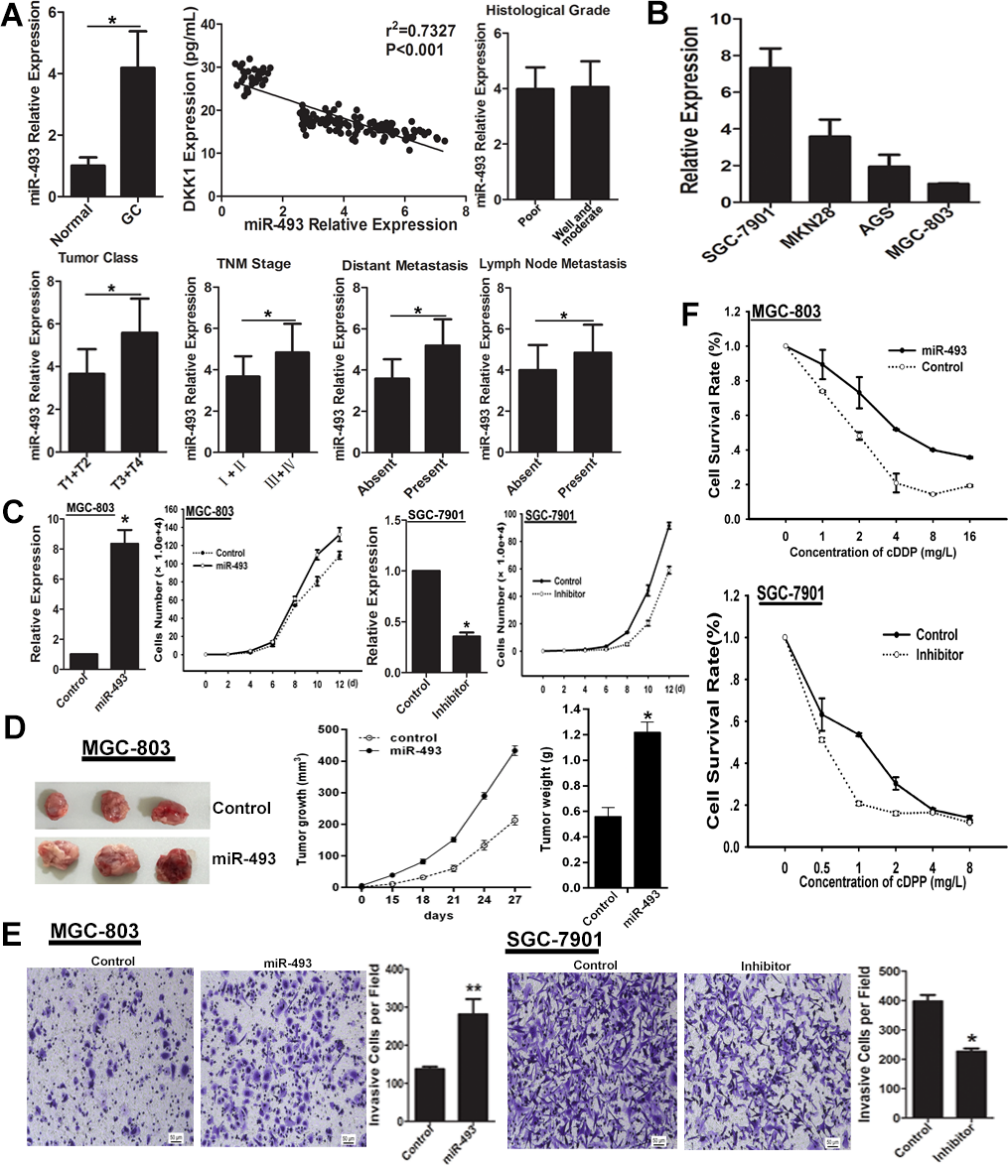
miR-493 promotes GC cells proliferation, invasion and chemo-resistance (**A**) The expression pattern of miR-493 in serum of GC patients and normal control was evaluated by real-time RT-PCR. The expression correlation between DKK1 and miR-493 was analyzed in serum specimens of GC patients and normal controls. Furthermore, the distribution of miR-493 in serum of GC patients with different clinicopathologic parameters was also analyzed. *p < 0.01. (**B**) The level of miR-493 expression in different GC cell lines was detected by real-time RT-PCR. (**C**) The expression level of miR-493 was determined after expression interference, vs control, *p < 0.01. The role of miR-493 on cell proliferation was determined and the average cell number of each time point was indicated. (**D**) Tumor growth was monitored every 3 days. Tumor size and weight were recorded. Data are represented as a mean ± SD from three mice. *vs* control, *p < 0. 01. (**E**) The invasive potential was determined by transwell assay. vs related control, *p < 0.05, **p < 0.01. (**F**) miR-493 overexpression decreased the sensitivity of GC cells to cDDP, but miR-493 knockdown enhanced the chemo-sensitivity of GC cells.

To assess the potential role of miR-493 in GC, we evaluated the expression pattern of miR-493 in GC serum and GC cells by real-time RT-PCR. The results indicated that miR-493 was up-regulated in GC serum compared to normal control group (Figure 4A). Correlation analysis showed that DKK1 level inversely correlated with miR-493 level in GC serum (Figure 4A). In the 116 serum samples of GC patients, miR-493 level was positively correlated with clinical parameters, such as tumor class, TNM stage, distant metastasis and lymph node metastasis, but not histological grade (Figure 4A). As shown in Figure 4B, miR-493 was highly expressed in SGC-7901 cell line with high-invasive ability but lowly expressed in low-invasive GC cell line MGC-803. Additionally, we found miR-493 overexpression promoted the cell proliferation, and miR-493 functional inhibition got a contrary result (Figure 4C). Importantly, the effect of miR-493 on growth in GC cells was further examined in nude mice with GC xenografts. We injected subcutaneously into the oxter of Athymic mice with miR-493 overexpressing MGC-803 cells and their control cells. As shown, tumors derived from miR-493 overexpressing MGC-803 cells had a significantly increased tumor size compared with related control cells (Figure 4D). Similarly, transwell assay showed that miR-493 markedly enhanced the invasion of GC cells (Figure 4E). Furthermore, we found miR-493 decreased the chemo-sensitivity of GC cells to cDDP, and miR-493 inhibitor enhanced the chemo-sensitivity (Figure 4F). Collectively, these data indicated that miR-493 is highly expressed in GC, and promotes the proliferation, invasion and chemo-resistance of GC cells.

### DKK1 reverses the role of miR-493 in GC

**Figure 5:**
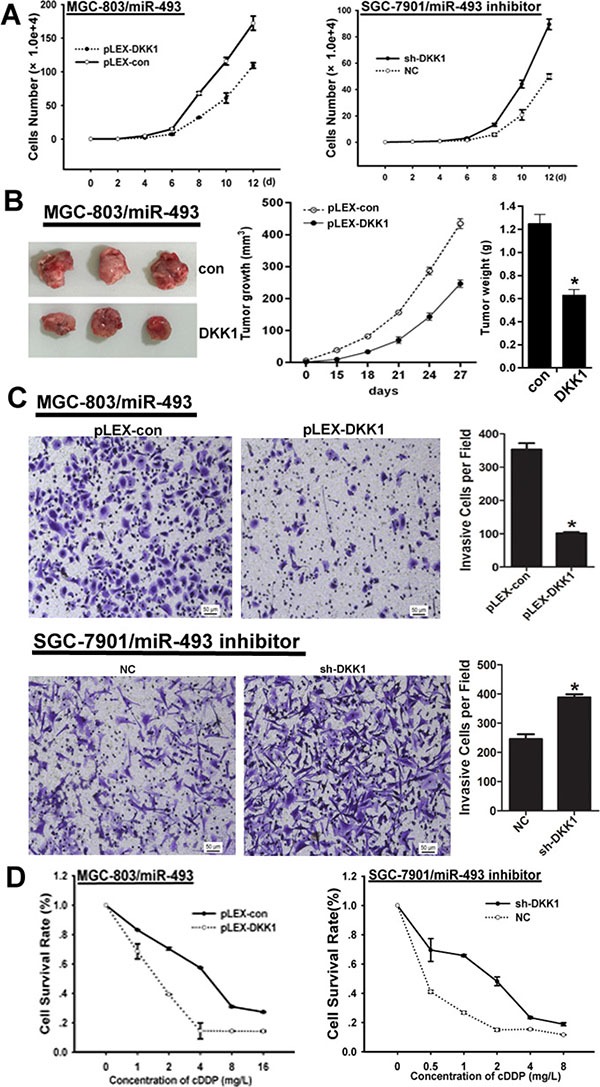
DKK1 reverses the function of miR-493 in GC cells (**A**) DKK1 reverses the effect of miR-493 on proliferation in GC and the average cell number of each time point was indicated. (**B**) DKK1 reverses the effect of miR-493 on GC cells growth *in vivo*. Tumor growth was monitored every 3 days. Tumor size and weight were recorded. Data are represented as a mean ± SD from three mice. *vs* control, *p < 0. 01. (**C**) DKK1 abrogates the effect of miR-493 on invasion and the invasive potential was determined by transwell assay. *vs* related control, *p < 0.05, **p < 0.01. (**D**) DKK1 impairs the effect of miR-493 on chemo-resistance in GC cells determined by MTS assay.

To investigate whether the tumor promotive effect of miR-493 was mediated by repressing DKK1 expression, we explored the rescue function of DKK1 in GC cells with different level of miR-493. Here, our findings indicated that DKK1 could reverse the promotive effect of miR-493 on proliferation in MGC-803 cells, but DKK1 knockdown could abrogate the suppressive effect of miR-493 inhibitor on proliferation in SGC-7901 cells (Figure 5A). Additionally, ectopic DKK1 expression impaired the promotive effect of miR-493 on tumor growth of MGC-803 *in vivo* (Figure 5B). Moreover, DKK1 also inhibit the invasive potential mediated by miR-493 in GC cells (Figure 5C). As shown in Figure 5D, overexpressed DKK1 reversed the chemo-resistance induced by miR-493 in GC cells. These results suggested miR-493 acts as a tumor promoter in GC via modulating DKK1 expression.

## DISCUSSION

Most GC patients present with locally advanced or metastatic disease at the time of diagnosis and experience resistance to anticancer therapy and poor overall prognosis. Thus, there is urgent need to elucidate the precise molecular mechanisms involved in growth, invasion, metastasis and chemo-resistance of GC for improving current treatments in the clinic. In the present study, DKK1 expression in GC was decreased and inversely correlated with miR-493 expression. Bioinformatics analysis combined with experimental validation revealed that DKK1 is a novel direct target of miR-493 in GC cells. MiR-493 promotes GC progression via suppressing DKK1 expression.

DKK1 had been reported to be highly expressed in several cancers and negatively correlated with prognosis. It had been demonstrated that elevated DKK1 expression was an early event in prostate cancer (PCa), but during PCa progression, DKK1 expression declined, particularly in advanced bone metastases. High DKK1 expression within PCa metastasis was further associated with shorter overall survival [24]. It was also characterized that high DKK1 serum levels were associated with a poor survival in patients with PCa [25]. The significance of DKK1 as a serum protein marker for HCC and non-small cell lung cancer (NSCLC) has been investigated [26, 27]. In apparent contradiction to the reports stated above, DKK1 expression was showed to be decreased in multiple cancers, including cancer types from stomach, colon, ovary and kidney [17, 23]. The serum DKK1 levels were lower in NSCLC and SCLC patients without any treatment, but after surgery and/or chemotherapy treatment, DKK1 expression level elevated [28]. So, DKK1 levels increased in serum from cancer patients whether resulted from treatment interferences remain unclear. In this study, we found the DKK1 levels in serum and tissues from GC patients without any treatment were decreased. DKK1 expression was negatively related with clinical parameters, such as tumor class, TNM stage, distant metastasis and lymph node metastasis, but positively related with prognosis. We also found cDDP could up-regulate DKK1 expression in GC cells (data was not shown), together with the evidence provided by Xu *et al.* [28], we proposed that surgery and/or chemotherapy treatment would increase DKK1 expression *in vivo* and vitro. For further study, DKK1 level in GC patients before and after treatment should be analyzed.

In regard to mechanisms for DKK1 down-regulation in GC, we focused on microRNAs (miRNAs). MiRNAs are a class of endogenous, non-coding single-strand small molecular RNAs. MiRNAs have been involved in many cellular processes, such as proliferation, differentiation, apoptosis and so on. MiRNAs induce posttranscriptional gene silencing by tethering RISC (RNA-induced silencing complex) to partly complementary sequence motifs in the 3′UTR of target mRNAs [29, 30]. Increasing evidences suggested that miRNAs were abnormally expressed in many kinds of cancers, functioning as oncogenes or tumor suppressors [31]. Previous work has demonstrated that miR-493 functions as a tumor suppressor. It was found that miR-493 expression decreased in bladder cancer and overexpressed miR-493 suppressed bladder cancer cells motility through down-regulation of RhoC and FZD4 [32]. Through dropout screening in a mouse model of liver metastasis, it was demonstrated that miR-493 induced cell death of metastasized cells, and inhibited liver metastasis of colon cancer cells by directly targeting IGFIR [33]. Gu *et al.* found that the expression of miR-493 was markedly reduced in pulmonary carcinoma and ectopic expression of miR-493 impaired cell growth and invasion *in vitro* and vivo through directly targeted E2F1 [34]. But the opposite results were detected in GC here. We found that the serum level of miR-493 was up-regulated in GC patients, which was inversely correlated with DKK1 level. Bioinformatics analysis together with luciferase reporter assay proved that miR-493 targeted DKK1 through directly binding to its 3′UTR. MiR-493 functioned as tumor promoter in GC via targeting DKK1 expression. Whether miR-493 exerts its role in cancer with cell-context dependence should be further explored.

Summary, we here demonstrated that DKK1 acted as a tumor suppressor in GC, and its expression level was correlated with GC prognosis. Meanwhile, miR-493 played an oncogenic role in GC by directly targeting *DKK1*. Our results provide new insight into the role of miR-493/DKK1 signaling pathway in GC progression and suggest potential molecular target for the treatment of GC.

## MATERIALS AND METHODS

### Clinical specimens

116 cases of GC patients' serum specimens without surgery and/or chemotherapy treatment, and 30 cases of normal patients' serum specimens were collected from Cancer Center of Guangzhou Medical University. The blood samples were centrifuged 10 min, 800 g at 4°C, then the supernatant was transferred into a new tube, and the serum was frozen at −80°C until use. All data, including histological grade, tumor class, TNM stage, distant metastasis and lymph node metastasis were obtained from clinical and pathologic records. The GC tissue samples were collected from two separate cohorts. For the first cohort, 52 cases of matching GC tissue samples were obtained from the patients whose serum DKK1 level had been determined. For the second cohort, additional 80 patients with consecutive GC from Cancer Center of Guangzhou Medical University were enrolled in the study to undergo immunohistochemistry and long-term survival analysis. Follow-up information, including overall survival was also collected. The study was approved by the ethics committee of Cancer Center of Guangzhou Medical University.

### Cell culture

GC cell lines SGC-7901, MKN28, AGS and MGC-803 were all obtained from the American Type Culture Collection. Cells were cultured in RPMI-1640 (Life Technologies) with 10% fetal bovine serum (Life Technologies) in a humidified cell incubator with an atmosphere of 5% CO_2_ at 37°C.

### RNA extraction and real-time RT-PCR

Total RNA was isolated from treated cells using TRIzol Reagent (Life Technologies), and then RervertAid First Strand cDNA Synthesis Kit (Ferments Life Science) was used to synthesize cDNA. Real-time RT-PCR for *DKK1* detection was performed using SYBR Select Master Mix (Life Technologies) following the manufactory instruction. *GAPDH* was used as an internal control. The primers for *GAPDH* were: forward primer 5′-ATTCCATGGCACCGTCAAGGCTGA-3′, reverse primer 5′-TTCTCCATGGTGGTGAAGACGCCA-3′; for *DKK1* were: forward primer 5′-CCTTGAACTC GGTTCTCAATTCC-3′, reverse primer 5′-CAATGGTCT GGTACTTATTCCCG-3′. The gene expression threshold cycle (CT) values of *DKK1* were calculated by normalizing with internal control *GAPDH* and relative quantization values were calculated.

miRNAs were purified from serum specimens by miRCURY^™^ RNA Isolation Kit-Biofluids (EXIQON), cDNA was generated with the miScript II RT Kit (QIAGEN) and real-time RT-PCR was done by miScript SYBR Green PCR Kit (QIAGEN) following the manufacturer's instructions. Primers for miR-493 and endogenous control RNU6 were purchased from QIAGEN. The gene expression threshold cycle (CT) values of miRNAs were calculated by normalizing with internal control RNU6 and relative quantization values were calculated.

### Transfection

Cells were trypsinised, counted and seeded onto 6-well plates the day before transfection to ensure 70% cell confluence on the day of transfection. The transfection of pLEX-DKK1, psi-LVRU6GP-shDKK1 and pEZX-miR-493 plasmids and related control plasmids were carried out using Lipofectamine 2000 (Invitrogen) in accordance with the manufacturer's procedure. Related stable cell lines were selected with puromycin treatment. The miR-493 inhibitor and control (Exiqon) with a final concentration of 100 nM were also transfected with Lipofectamine 2000. For proliferation assay, the miR-493 inhibitor and control were transfected every 3 days. The efficiency of transfection was determined by followed western blot and real-time RT-PCR.

### Luciferase reporter assay

The DNA sequences with each 50 base at up-and downstream of miR-493 binding site in the 3′UTR of DKK1 (as wild-type version) and DNA sequences with 7 bases deleted in the miR-493 binding site (as mutant version), were synthesized with restriction sites for SpeI and HindIII located at both ends of the oligonucleotides for further cloning, and subsequently cloned into pMir-Report plasmid downstream of firefly luciferase reporter gene. Cells were seeded in 96 well-plates and co-transfected with pMir-Report luciferase vector, pRL-TK Renilla luciferase vector and pEZX-miR-493 or miR-493 inhibitor using Lipofectamine 2000. After transfection of 48 h, luciferase activity was determined using a Dual-Luciferase Reporter Assay System (Promega) on the BioTek Synergy 2. The Renilla luciferase activity was used as internal control and the firefly luciferase activity was calculated as the mean ± SD after being normalized by Renilla luciferase activity.

### Western blot

Total protein was harvested by RIPA lysis buffer (Pierce) with Protease Inhibitor Cocktail (Pierce), and quantified using the BCA protein assay kit (Pierce). These protein was separated by SDS-PAGE gel and transferred onto PVDF membrane (Millipore), blocked with Tris buffer contained 0.1% Tween-20 and 5% nonfat milk at 4°C. The membranes were incubated with anti-DKK1 (Novus Biologicals, 1:2000 dilution; anti-GAPDH, Santa Cruz biotechnology, 1:500), and followed by HRP-conjugated secondary antibody (Merk, 1:5000), respectively. The signal was detected using ECL detection system (Millipore) as described by the manufacturer.

### Proliferation assay

Related cells were seeded into 24-well plates at a density of 1000 cells per well. Every two day, the cells number of independent three wells was counted, and the average number was calculated. The cell proliferation assay was performed on 0, 2, 4, 6, 8, 10 and 12 days, the average cell number of each time point was noted.

### MTS assay

Cells were seeded into 96-well plates, and different concentration of cDDP was added into each well. Approximately 48 h after treatment, 20 μL MTS (promega) was added into each well, and the plate was incubated at 37°C for 4 h. The absorbance at 490 nm of each well was recorded by microplate reader BioTek Synergy 2. Growth rate was calculated as the ratio of the absorbance of the experimental well to that of the control.

### Transwell assay

Invasion of cells was evaluated with the Cell Invasion Assay Kit (BD Biosciences) following the manufacturer's instructions. Briefly, at 36 h post-transfection, 3 × 10^4^ cells in 300 μL serum-free medium were added to the upper chamber precoated with ECMatrix^™^ gel. And 0.5 mL medium with 10% FBS was added to the lower chamber as a chemoattractant. Subsequently, cells were incubated at 37°C for 24 h. Furthermore, non-invading cells were removed with cotton swabs. In addition, cells that migrated to the bottom of the membrane were fixed with pre-cold methanol and stained with 2% Giemsa solution. Finally, Stained cells were visualized under a microscope. To minimize the bias, at least three randomly selected fields with 100 × magnification were counted, and the average number was taken.

### Enzyme-linked immunosorbent assay (ELISA)

The serum concentration of DKK1 was measured by Human DKK1 Quantikine ELISA Kit (R & D system) according to the manufacturer's instructions. Briefly, 100 μL Assay Diluent was added into each well, followed by 100 μL standard, control or sample and the microplate was incubated at room temperature for 2 h. Then each well was washed 3 times, blocked with 200 μL Conjugate and incubated for 2 h at room temperature, followed by another washing step. In addition, 200 μL Substrate Solution was added and incubated at 37°C for 30 mins without light. Finally, the reaction was terminated by adding 50 μL Stop Solution. The absorbance was read at a 450 nm wavelength. The concentration of DKK1 was evaluated based on standard curves.

### Immunohistochemistry

All tissue samples were embedded in paraffin, and immunostained with DKK1 antibody after a microwave antigen retrieval procedure was performed. Furthermore, samples were orderly incubated with biotin labeled secondary antibody, DAB detection system and hematoxylin. In addition, the extent of the staining was visually evaluated on a scale of 1 (no staining) to 4 (strong staining). Approximately 800 cells were evaluated for each sample by two observers, and the mean value for each staining was calculated. Clinical samples were divided into high-expression or low-expression groups based on DKK1 expression scores.

### Statistical analysis

All data was presented as means ± standard deviation from three independent experiments. SPSS19.0 software was used to carry out statistical analysis. The differences between groups were investigated by student's *t*-test with only two groups or one-way analysis of variance (ANOVA) when more than two groups were compared. *P* < 0.05 was considered statistically significant.
